# Animal models for Ebola and Marburg virus infections

**DOI:** 10.3389/fmicb.2013.00267

**Published:** 2013-09-05

**Authors:** Eri Nakayama, Masayuki Saijo

**Affiliations:** Department of Virology 1, National Institute of Infectious DiseasesTokyo, Japan

**Keywords:** Ebola virus, Marburg virus, filovirus, animal models, viral hemorrhagic fever

## Abstract

Ebola and Marburg hemorrhagic fevers (EHF and MHF) are caused by the Filoviridae family, *Ebolavirus* and *Marburgvirus* (ebolavirus and marburgvirus), respectively. These severe diseases have high mortality rates in humans. Although EHF and MHF are endemic to sub-Saharan Africa. A novel filovirus, Lloviu virus, which is genetically distinct from ebolavirus and marburgvirus, was recently discovered in Spain where filoviral hemorrhagic fever had never been reported. The virulence of this virus has not been determined. Ebolavirus and marburgvirus are classified as biosafety level-4 (BSL-4) pathogens and Category A agents, for which the US government requires preparedness in case of bioterrorism. Therefore, preventive measures against these viral hemorrhagic fevers should be prepared, not only in disease-endemic regions, but also in disease-free countries. Diagnostics, vaccines, and therapeutics need to be developed, and therefore the establishment of animal models for EHF and MHF is invaluable. Several animal models have been developed for EHF and MHF using non-human primates (NHPs) and rodents, which are crucial to understand pathophysiology and to develop diagnostics, vaccines, and therapeutics. Rhesus and cynomolgus macaques are representative models of filovirus infection as they exhibit remarkably similar symptoms to those observed in humans. However, the NHP models have practical and ethical problems that limit their experimental use. Furthermore, there are no inbred and genetically manipulated strains of NHP. Rodent models such as mouse, guinea pig, and hamster, have also been developed. However, these rodent models require adaptation of the virus to produce lethal disease and do not mirror all symptoms of human filovirus infection. This review article provides an outline of the clinical features of EHF and MHF in animals, including humans, and discusses how the animal models have been developed to study pathophysiology, vaccines, and therapeutics.

## Introduction

**Figure 1:**
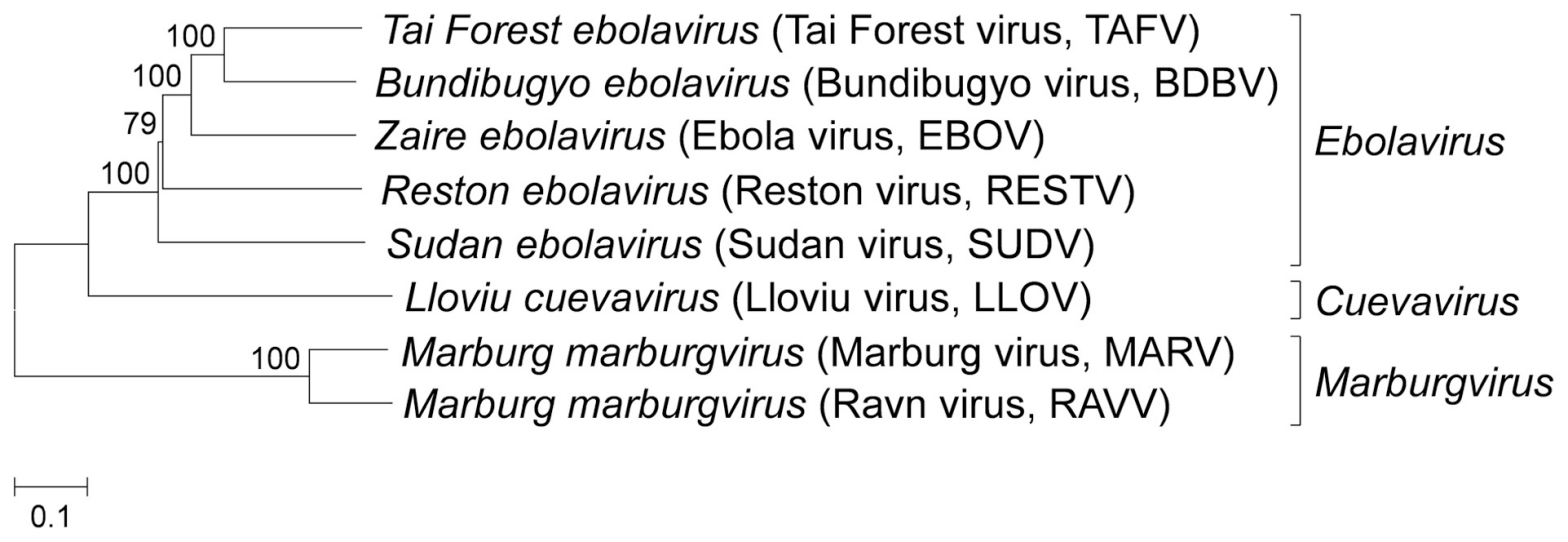
**Phylogenetic analysis of filovirus based on nucleotide sequence.** The phylogenetic tree based on complete viral genome sequences was constructed by using the neighbor-joining method. Numbers at branch points indicate bootstrap values (1000 replicates). The GenBank accession numbers of Tai Forest virus (TAFV), Bundibugyo virus (BDBV), Ebola virus (EBOV), Reston virus (RESTV), Sudan virus (SUDV), Lloviu virus (LLOV), Marburg virus (MARV), and Ravn virus (RAVV) are FJ217162, FJ217161, AF086833, AB050936, AY729654, JF828358, DQ217792, and EF446131, respectively.

The Family *Filoviridae* includes three accepted genera, *Ebolavirus* (ebolavirus), *Marburgvirus* (marburgvirus), and *Cuevavirus* (Figure 1) (Kuhn et al., 2011, 2013). Filoviruses are classified as biosafety level 4 (BSL-4) agents because they cause severe hemorrhagic fevers in humans and non-human primates (NHPs) with high case-fatality rates, ranging between 23 and 90% (Sanchez et al., 2007). Each of the *Marburgvirus* and *Cuevavirus* genera consists of a single species, *Marburg marburgvirus* and *Lloviu cuevavirus*, respectively. The genus *Marburgvirus* has two subspecies: Marburg virus (MARV) and Ravn virus (RAVV). The genus *Ebolavirus* is divided into five distinct species, *Zaire ebolavirus* (Ebola virus, EBOV), *Sudan ebolavirus* (Sudan virus, SUDV), *Tai Forest ebolavirus* (Tai Forest virus, TAFV), *Bundibugyo ebolavirus* (Bundibugyo virus, BDBV), and *Reston ebolavirus* (Reston virus, RESTV; Kuhn et al., 2013). EBOV is highly virulent to humans and NHPs with a mortality rate of up to 90% in African epidemics. The case fatality rate of SUDV and BDBV is ~50 and 25%, respectively; the only person known to have been infected with TAFV survived. RESTV has been known to cause symptomatic disease in NHPs but not in humans. Lloviu virus belonging to the genus *Cuevavirus* was identified in the absence of replicating isolates during an investigation of die-off bats in Spain and the virulence for humans and NHPs has not been assessed (Negredo et al., 2011).

Although there has been an increasing frequency of filovirus outbreaks reported from endemic regions of Africa and Asia in recent years, there are no licensed vaccines or effective therapeutics for filovirus hemorrhagic fever.

The primary source of patients with filovirus hemorrhagic fever was mainly linked to exposure to animal carcasses found in the forest or to the putative bat reservoir, resulting in subsequent transmission through direct person-to-person contact (Leroy et al., 2004, 2009). Filoviruses enter the body via direct contact with infectious blood and/or body fluids. After an incubation period of 2–21 days, non-specific initial symptoms such as fever, chills, fatigue, headache, and myalgia appear. About 5–7 days after onset, a maculopapular rash usually develops on the face, buttocks, trunk, and/or arms and later generalizes over the entire body. As disease progresses, systemic (prostration, lethargy), gastrointestinal (anorexia, vomiting, abdominal pain, diarrhea), respiratory (chest pain, breath shortness, cough, nasal discharge), vascular (conjunctival injection, postural hypotension, edema), and neurological (headache, confusion, coma) manifestations are observed. Some patients develop multiple foci of mucosal hemorrhage, which is especially evident in conjunctiva and gingiva together with bleeding from venipuncture sites. Hemorrhagic symptoms observed during the peak of the illness include petechiae, ecchymoses, epistaxis, mucosal hemorrhages, and/or visceral hemorrhagic effusions. In fatal cases, patients die with hypovolemic shock and multiple organ failure between Day 6 and 16.

Animal models of filovirus infection have been developed in mice, guinea pigs, hamsters, and NHPs (Connolly et al., 1999; Bente et al., 2009; Bradfute et al., 2012; Wahl-Jensen et al., 2012). The development of animal models that accurately reflect human disease is critical to understanding the pathogenesis of Ebola and Marburg hemorrhagic fevers (EHF and MHF, respectively), because filoviral outbreaks in humans are sporadic and there is limited clinical data and access to human tissue. Since the wild-type virus replicates to high titers in NHPs and the virus causes symptoms, including hemorrhage and shock, which are similar to those of patients with EHF and MHF, NHP models may be the most useful to evaluate the efficacy of candidate vaccines and treatment measures. However, small animal models are also needed for preliminary evaluation of vaccines and therapeutic interventions against filovirus diseases, because of the ethical and handling issues related to using NHPs.

Here, we summarize and discuss the animal models developed for the study of hemorrhagic fever caused by filoviruses.

## Mouse models

In contrast to the development of the NHP and guinea pig models, as described in later sections, development of a mouse model of filovirus infection has been unsuccessful due to the fact that adult immunocompetent mice were resistant to wild-type filovirus infection. The intraperitoneal or intracerebral inoculation of newborn mice and 4-day-old suckling mice with non-mouse adapted EBOV was shown to cause lethal infections, but 8-day-old or older mice did not show any symptoms (Johnson et al., 1995; Bray, 2001). Serial passage of wild-type EBOV in suckling mice was needed for adaptation, in which the virus acquired lethal virulence to adult immunocompetent mice (Bray, 2001). Intraperitoneal inoculation of mouse-adapted EBOV with a 1–100 plaque forming unit (pfu) dose (30–3000 times the median lethal dose) caused lethal infection to 5-week-old BALB/c, C57BL/6 and ICR (CD-1) mice, but subcutaneous inoculation of the virus at a dose of 10^6^ pfu did not cause symptomatic illness in 3-week-old adult mice (Bray, 2001). This phenomenon is not observed in NHP and guinea pig models, which are susceptible to wild-type EBOV infection through any route of inoculation. CD8^+^ T cells and perforin, but not B cells and CD4^+^ T cells, are required for resistance to subcutaneous inoculation of EBOV (Gupta et al., 2005). It is supposed that the presence of regional lymph nodes and/or Langerhans cells in the skin contributes to protection from filoviral subcutaneous infection via activation of CD8^+^ cells, however, there are no reports to prove this hypothesis.

It has been shown that mouse-adapted filovirus is fatal over a broad range of ages in BALB/c mice. Infected mice became acutely ill with symptoms of ruffled fur, reduced activity, and weight loss on Day 3–4 post-infection and died on Days 5–7, although these lengths differed depending on the challenge dose (Bray et al., 1999; Warfield et al., 2009). Virus titer in the liver and spleen exceeded 10^7^ pfu/g within 3 days after infection and then reached a maximum of over 10^9^ pfu/g at Day 5 post-inoculation. These titers exceeded the peak viral concentrations in the liver and spleen of infected guinea pigs (about 10^6^ pfu/g) and NHPs (about 10^7^ pfu/g; Bray et al., 1999). The virus is generally undetectable in serum on Day 1, but by Day 3 the viremia level peaks at approximately 10^7^ pfu/ml, which was comparable to that in NHPs and exceeds that in guinea pigs (10^4−5^ pfu/ml; Bray et al., 1999).

As seen in the NHP model, the systemic viral spread results in extensive infection and necrosis of the liver, spleen, and other organs (Bray et al., 1999; Warfield et al., 2009). In liver from mice infected with mouse-adapted EBOV or RAVV, viral replication was observed in hepatocytes, Kupffer cells, and sinusoidal endothelial lining cells. Histological lesions were observed by Day 4 after inoculation, including coalescing, foci of hepatocellular vacuolar change, degeneration, and necrosis of hepatocytes. In the spleen, viral antigen was detected on Day 2 after infection, at which point coagulopathy, such as disseminated intravascular coagulation (DIC) accompanied by prolongation of prothrombin time (PT) and activated partial thromboplastin time (aPTT), was not observed in the moribund mice (Bray et al., 2001; Warfield et al., 2009).

Mouse-adapted EBOV initially infects macrophages and other mononuclear phagocytes at the site of invasion and in regional lymph nodes. The major target cells of infection are as the same as those in humans, NHPs, and guinea pigs (Davis et al., 1997; Connolly et al., 1999; Zaki and Goldsmith, 1999; Gibb et al., 2001). Viral replication in mononuclear phagocytes in the lymph node, spleen, and thymus and an increase in the number of virus-infected Kupffer cells in the liver were observed by Day 3 after infection. Most mononuclear phagocytes throughout the body appear to be infected and the mice died by Days 5–6.

The adaptation of EBOV to adult mice resulted in 8 amino acid changes in both the coding and non-coding regions of the virus genome compared to the original wild-typed virus (Ebihara et al., 2006). Nucleotide substitutions leading to amino acid changes were found in VP35, VP24, NP, and L viral proteins. VP24 and VP35 are known as type I interferon (IFN) antagonists and interfere with type I IFN-mediated antiviral response *in vitro* (Bowen et al., 1980; Basler et al., 2000; Bente et al., 2009; Halfmann et al., 2011). VP24 functions as an IFN antagonist by binding karyopherin α and blocking nuclear accumulation of signal transducer and activator of transcription 1 (STAT1; Reid et al., 2007). VP35 is also implicated in blocking type I IFN responses by inhibiting phosphorylation of interferon regulatory factor (IRF) 3 and 7 by the Tank binding kinase-1 and I-Kappa-B kinase epsilon, and sequestering the viral RNA from detection by RIG-I like receptor (Ramanan et al., 2011, 2012). It is considered that there is a significant relationship between filoviral virulence and the ability of the virus to evade the type I IFN-induced antiviral response (van der Groen et al., 1979). The mutations in NP and VP24 genes were found to be critical for acquisition of EBOV virulence in adult mice, but not VP35 mutations (Ebihara et al., 2006). NP is tightly coupled with viral RNA and forms the nucleocapsid complex together with L, VP30, and VP35. Although it is unclear how NP is involved in the IFN response, NP is thought to confer evasion from the IFN-stimulated antiviral responses directly or indirectly in infected mice.

The mutations identified for adaptation of marburgvirus to mice differed from those required for that of ebolavirus. The amino acid mutations were found in VP40, VP35, NP, and VP30 in mouse-adapted RAVV compared to those of the wild-type virus derived from a patient (Warfield et al., 2009). It is still unclear what is the role and necessity of each of the mutations in mouse-adapted RAVV. Further experiments are required to clarify which mutations are critical for adaptation to mice.

Adult mice treated with antibodies against IFN-α/β became susceptible to infections with non-adapted EBOV or SUDV infected via the intraperitoneal route, and to mouse-adapted virus infected via the subcutaneous route (Bray, 2001). Furthermore, non-adapted EBOV, SUDV, MARV, or RAVV caused illness in KO mice lacking type I IFN receptors or the STAT1 protein (Bray, 2001). These results suggest that inhibition of type I IFN response against filovirus infections is critical for pathogenesis in mouse models.

Mice infected with the mouse-adapted filovirus are different from humans and NHPs infected with the original filoviruses in terms of a lack of severe coagulation disorder and fibrin deposition. Mouse models are useful tools for studying basic aspects of replication, pathogenesis, and immune responses and also serve as an irreplaceable platform for evaluating the efficacy of the wide range of the candidate vaccines and therapeutic agents.

## Guinea pig models

Guinea pigs are susceptible to several arenaviruses, Lassa fever virus, Junin virus, and Guanarito virus and used as animal models for human viral hemorrhagic fevers caused by these viruses (Bowen et al., 1977; Jahrling et al., 1982; Kenyon et al., 1990; Hall et al., 1996). However, infection of guinea pigs with wild-type filovirus usually causes only a transient febrile illness (Simpson et al., 1968; Robin et al., 1971; Bowen et al., 1977). Filoviruses need to be serially passaged in guinea pigs to acquire the ability to cause lethal infection in guinea pigs (Simpson et al., 1968; Robin et al., 1971; Ryabchikova et al., 1996). Guinea pigs inoculated with guinea pig (GP)-adapted virus showed similar symptoms such as fever, anorexia, and dehydration, to those reported in humans and NHPs infected with wild-type filovirus (Simpson et al., 1968; Robin et al., 1971; Connolly et al., 1999). GP-adapted EBOV-infected guinea pigs showed fibrin deposition coincident with decreases in platelet count during the late stage of infection (Connolly et al., 1999). GP-adapted EBOV replicated to high titers in the spleen, liver, adrenal gland, and lung, resulting in viremia in guinea pig models, although the peak titers were less than those demonstrated in NHPs. Viremia in guinea pigs developed within 2 days after inoculation and increased during the course of the disease, reaching a peak on Day 7 (>10^4^ pfu/ml; Connolly et al., 1999; Subbotina et al., 2010). The guinea pigs infected with the GP-adapted filovirus died on 7–9 days after infection (Simpson et al., 1968; Robin et al., 1971; Connolly et al., 1999; Subbotina et al., 2010).

Histopathological changes in the liver of guinea pigs infected with GP-adapted filoviruses included replication of the viruses in Kupffer cells, multifocal necrosis of hepatocytes, and congestion and destruction of the sinusoid wall, which were also similar to those reported in humans and NHPs infected with wild-type filoviruses (Korb and Slenczka, 1971; Connolly et al., 1999; Ryabchikova et al., 2003). However, infiltrations of inflammatory cells in the liver and other organs were mild or absent (Connolly et al., 1999; Ryabchikova et al., 2003). Lymphoid necrosis was observed in the spleen and lymph nodes of guinea pig models.

Neutrophilia and lymphopenia became detectable in the guinea pig model as early as 2 days after infection and the severity continued to increase over the course of infection (Connolly et al., 1999; Subbotina et al., 2010). However, lymphocyte bystander apoptosis, an important feature in NHPs and mice, was not prominent in guinea pigs (Bray et al., 1999; Connolly et al., 1999; Bradfute et al., 2007). Thrombocytopenia was marked during the late stages of the disease when guinea pigs became moribund and platelets fell from a mean of ~500,000 to <50,000/μl. Fibrin deposition was a late event, beginning only modestly in the liver and spleen on Day 4, with increases in distribution and amount on Days 7–9, coincident with decreases in platelet counts.

Comparative sequence analysis of the complete genomes of the GP-adapted EBOV and wild-type virus showed 8 nucleotide differences, which led to 5 amino acid substitutions; single amino acid mutations in NP and L and 3 mutations in VP24 (Volchkov et al., 2000). Using a reverse genetics approach, it was shown that VP24 had a critical role in the pathogenesis and the amino acid changes in VP24 were essential to achieve EBOV virulence in guinea pigs. VP24 was demonstrated to antagonize IFN signaling by binding host karyopherin α proteins and prevent transport of the tyrosine phosphorylated transcription factor STAT-1 to the nucleus (Reid et al., 2007; Mateo et al., 2010; Zhang et al., 2012). One of the substitutions in VP24 of GP-adapted EBOV was located in the proximal domain, which was recently shown to be involved in karyopherin binding and required for efficient control of the IFN response (Mateo et al., 2010). However, the mutations associated with EBOV adaptation to the guinea pigs did not affect the ability of VP24 to inhibit IFN signaling (Mateo et al., 2011). VP24 participates in the assembly and/or proper formation of viral nucleocapsids. The lack of virulence of wild-type virus in guinea pigs was associated with an inability of the virus to replicate in and/or be released from hepatocytes and macrophages efficiently (Mateo et al., 2011). Wild-type VP24 is somehow incapable of participating in assembly of viral nucleocapsids in guinea pigs. Mutations in VP24 for adaption to guinea pigs recovered the ability of EBOV to replicate in both macrophages and hepatocytes and to facilitate the systemic spread of the virus.

## Syrian golden hamster models

The pathogenesis of rodent-adapted filoviruses differs in some aspects from those of humans and NHPs infected with wild-typed virus (Table 1). Fever and cutaneous rash, which are major clinical signs of EHF and MHF in humans and NHPs, are absent in mice infected with mouse-adapted virus. Fever appears in guinea pigs infected with GP-adapted virus, but rash does not develop in these animals. Mice infected with mouse-adapted virus do not consistently display coagulation abnormalities. Compared to mice, guinea pigs infected with GP-adapted virus develop coagulation defects, including a drop in platelet counts and an increase in coagulation time, but coagulopathy (i.e., DIC) are not as marked as that observed in NHPs. Furthermore, lymphocyte apoptosis observed in humans, NHPs, and mice was not determined in the guinea pig model. Because of these differences in the rodent models, some vaccines (e.g., irradiated virion) and therapeutics (e.g., passive immunization with antiserum) that were effective in rodents challenged with adapted virus fail to protect NHPs challenged with wild-type virus (Wahl-Jensen et al., 2012).

**Table 1:** **Comparison of pathological features of different animal models of filovirus infection**.

	**Mouse**	**Guinea pig**	**Hamster**	**NHP**	**Human**
Virus adaptation	Adapted	Adapted	Adapted	Wild-type	Wild-type
Viremia	High	High	High	High	High^a^
Virulence	High	High	High	High	High^b^
Weight loss	Severe	Severe	No	Severe	Severe^c^
Rash	No	No	No	Yes	Yes^d^
Thrombocytpenia	Yes	Yes	Yes	Yes	Yes^e^
Lymphocyte apoptosis	Yes	Limited	Yes	Yes	Yes^f^
Cytokine response	Yes	Yes	Yes	Yes	Yes^g^
PT	Remained	Increased	Increased	Increased	Increased^h^
PTT/aPTT	Remained	Increased	Increased	Increased	Increased^i^
TT	ND	ND	Increased	Increased	ND
Fibrin deposition in organs	Little	Moderate	Little	Abundant	Observed^j^
Protein C activity	ND	ND	Decreased	Decreased	ND

a Ksiazek et al., 1999; Ndambi et al., 1999; Sanchez et al., 2004; Towner et al., 2004; Kuhn, 2008.

b Isaacson et al., 1978; Piot et al., 1978; Smith et al., 1978; Bwaka et al., 1999; Sanchez et al., 2007; Kuhn, 2008.

c Bwaka et al., 1999; Kuhn, 2008.

d Isaacson et al., 1978; Smith et al., 1978; Bwaka et al., 1999; Sanchez et al., 2007; Kuhn, 2008.

e Sanchez et al., 2007; Kuhn, 2008.

f Baize et al., 1999.

g Baize et al., 1999, 2002; Sanchez et al., 2007.

h Sanchez et al., 2007; Kuhn, 2008.

i Sanchez et al., 2007.

j Dietrich et al., 1978.

Abbreviations *PT* *prothrombin time* *PTT* *partial thromboplastin time* *APTT* *activated partial thromboplastin time* *TT* *thrombin time* *ND* *no data.*

Moreover, in the guinea pig model, the lack of available reagents and tools, such as quantitative reverse-transcription polymerase chain reaction (qRT-PCR) and ELISA for cytokine profiling, makes the guinea pig model less desirable. Therefore, the development of other rodent models that better recapitulate EHF and MHF in humans was desired for more relevant pathogenesis studies and high throughput screening of prophylactic and post-exposure treatment prior to their testing in NHPs.

Syrian golden hamster (*Mesocricetus auratus*) is broadly used in animal models for human infectious diseases (Zivcec et al., 2011). Suckling hamsters were susceptible to wild-type MARV but disease was evident in only 40–80% of the animals following either intracerebral or intraperitoneal inoculation (Zlotnik, 1971). The symptoms and the mortality which was up to 90% were observed by inoculation with ninth passage materials in 5–6-week-old hamsters. While the pathological changes in the hamsters are similar to those observed in other animal models including patients, encephalitis, which is not observed in other animals, were constantly demonstrated in all suckling hamsters, irrespective of route of inoculation, and in adult hamsters, when the virus was inoculated intracerebrally.

The Syrian golden hamster model was developed for EHF based on infection with mouse-adapted EBOV. Six-week-old hamsters infected intraperitoneally with 10^3^ focus forming unit (ffu) of mouse-adapted EBOV started to show clinical signs of disease, including ruffled fur and decreased activity, by Day 3 after infection and succumbed to disease within 4–5 days after infection. When inoculated subcutaneously, mouse-adapted virus failed to produce lethal infection in hamsters in the same way as the mouse model. Mouse-adapted EBOV-infected hamsters showed severe coagulopathy with prolonged PT, aPTT, and thrombin time (TT), in the late stage of infection (Ebihara et al., 2013). Other factors, including increased fibrinogen, decreased protein C activity, thrombocytopenia, and coagulation disorder were observed in the hamster model. The target organs were the mesenteric lymph node, spleen, liver, and adrenal gland. In the mesenteric lymph node, the target cells were the macrophages and dendritic cells (DCs). The viral antigens were found in macrophages, marginal reticular-like cells, and DC-like cells in the spleen, and Kupffer cells and hepatocytes in the liver. Histopathological changes, including inflammatory cell infiltration, cellular necrosis, and apoptosis, were mainly noted in the lymphoid organs (spleen and mesenteric lymph node) and liver. These pathological changes were similar to those demonstrated in NHPs and other rodent models (Baskerville et al., 1978; Fisher-Hoch et al., 1992; Jahrling et al., 1996; Ryabchikova et al., 1996, 1999; Bray et al., 1999; Connolly et al., 1999; Warfield et al., 2009). Fibrin deposits in liver sinuses, which are a hallmark of EBOV infection in humans and NHPs, were detected to a lesser extent in the hamster model (Ebihara et al., 2013). Suppression of type I IFN that enhances viral replication in target cells and contributes to lethal disease was observed (Ebihara et al., 2013).

It has been demonstrated that the mouse-adapted EBOV-based Syrian golden hamster model shows the most similar clinical and pathological features, including coagulation abnormalities, to those observed in humans and NHPs infected with wild-type EBOV.

## Non-human primate models

Although guinea pigs, mice, and hamsters models have been developed to study EHF and MHF as stated above, the rodent models are not ideal because mice and guinea pigs, except a novel hamster model, do not entirely exhibit coagulation disorders that are associated with human and NHPs filovirus infections (Table 1). Additionally the bystander death of large numbers of uninfected lymphocytes due to apoptosis that are hallmark features in filovirus-infected humans and NHPs is not present in infected guinea pigs. In mouse models, the bystander lymphocyte apoptosis was reported, but the process and morphology of lymphocyte apoptosis was different from those of filovirus-infecting humans and NHPs (Bradfute et al., 2007).

In NHP models, apoptosis was the primary reason for lymphocyte death, but the lymphocyte death in mouse models appeared to occur by apoptosis and apoptosis-like programmed cell death. Furthermore, NHPs are lethally infected with non-adapted filovirus isolates resulting in pathophysiology similar to that demonstrated in humans, although rodent models required serial passages of the virus for adaptation to produce lethal disease. Because of the aforementioned disadvantages and differences in the disease pathology, NHPs remain the most useful and reasonable model of EHF and MHF despite practical and ethical considerations leading to the restriction of experiments.

### Marburgvirus infection in NHPs

The first documented outbreak of MHF was associated with wild-caught African green monkeys (*Chlorocebus aethiops*) in Uganda and imported to Marburg and Frankfurt, Germany, and to Belgrade, Serbia Montenegro, former Yugoslavia, in 1967 (Martini, 1971). Since the first outbreak of MHF originated from the wild-caught African green monkeys, this species was an obvious choice for an animal model of MHF. At that time, rhesus macaques (*Macaca mulatta*) were found to be equally susceptible to infection and showed symptoms after inoculation with MARV (Hass and Maass, 1971). Cynomolgus macaques (*Macaca fascicularis*) were also well characterized as an MHF model (Hensley et al., 2011). After an incubation period of 2–6 days, the monkeys showed febrile illness, anorexia, diarrhea, skin rash, and hemorrhagic manifestations by any routes of MARV-inoculation (Simpson et al., 1968; Simpson, 1969; Murphy et al., 1971; Geisbert et al., 2007; Alves et al., 2010; Hensley et al., 2011). Death occurred by 6–13 days post-infection after a sudden decrease in body temperature and the mortality rate was almost 100%. It was shown that reducing the virus inoculum led to delayed onset of the disease and longer time to death without reduction in mortality rate (Hass and Maass, 1971). In the macaques, petechial rashes on the forehead, chest, axillae, and groins were prominent and resembled the rashes that appeared in patients with MHF, but intriguingly the rashes were not seen in African green monkeys (Simpson, 1969).

A marked lymphocytosis was observed at the beginning of the illness (Simpson et al., 1968; Simpson, 1969; Gonchar et al., 1991; Spiridonov et al., 1992; Johnson et al., 1996; Geisbert et al., 2007). Thrombocytopenia and leukocytosis due to increased neutrophilia were prominent on 5–6 days after infection (Hensley et al., 2011). Changes in coagulation systems, such as a decrease in circulating levels of protein C, an increase in levels of circulating D-dimer and fibrin deposition in tissues were noted at late stages of the disease (Geisbert et al., 2007; Hensley et al., 2011). The pathological changes in liver including multifocal necrosis of the parenchyma cells, and lymphocyte apoptosis in lymphoid tissues were prominent (Geisbert et al., 2000). Monocyte/macrophages and DCs in the lymphoid tissues as well as Kupffer cells and sinusoids lining cells in the liver were the primary target cells for infections with MARV (Hensley et al., 2011). The infection then progressed to parenchymal cells in the liver, adrenal gland, and high endothelial venules in lymphoid tissues. Finally, the infection spread to endothelial cells in a variety of organ tissues (Hensley et al., 2011). The virus or viral antigen was detected in liver, lymph nodes, spleen, adrenal grand, kidney, and blood in infected cynomolgus macaques. Onset of viremia occurred on Day 3, and in cynomolgus macaques and African green monkeys the maximum titer was 10^7−8^ pfu/ml on Day 8 after infection (Hass and Maass, 1971; Hensley et al., 2011).

Under experimental conditions, the possibility of aerosol transmission of MARV was shown in macaque models, although such a transmission route has not been described in human outbreaks (Pokhodiaev et al., 1991; Alves et al., 2010).

### Ebolavirus infection in NHPs

African green monkeys, cynomolgus macaques, rhesus macaques, and hamadryas baboons (*Papio hamadryas*) have been employed as a model of EBOV infection (Baskerville et al., 1978, 1985; Bowen et al., 1978, 1980; Ellis et al., 1978; Fisher-Hoch et al., 1985, 1992; Johnson et al., 1995; Jaax et al., 1996; Jahrling et al., 1996; Davis et al., 1997; Ryabchikova et al., 1999; Ignatiev et al., 2000; Geisbert et al., 2003b,e). The monkeys infected with EBOV became febrile 3 days after infection with temperatures above 40°C. Pyrexia usually persisted throughout the course of the disease, which usually ended in a decrease in temperature followed by death, which occurred within 5–8 days after infection (Baskerville et al., 1978; Bowen et al., 1978, 1980; Ellis et al., 1978; Fisher-Hoch et al., 1985; Luchko et al., 1995). By Day 4, anorexia developed with a loss of drinking ability, causing severe weight loss and dehydration. Some monkeys that survived until Day 5 had diarrhea, rectal bleeding, and/or intermittent melena. Petechial skin rashes appeared on the forehead, fore and hind limbs, and chest 4–5 days post-infection in macaques, but on Day 7 in baboons (Bowen et al., 1978; Ellis et al., 1978; Luchko et al., 1995; Ignatiev et al., 2000; Geisbert et al., 2003b). African green monkeys did not develop the cutaneous rash as demonstrated in monkeys infected with MARV (Simpson, 1969; Baskerville et al., 1978). Viremia became detectable within 3 days after infection with the maximum virus titer at the level of 10^6.5−7^ pfu/ml on Day 4–5 (Bowen et al., 1978; Fisher-Hoch et al., 1992; Jahrling et al., 1996; Geisbert et al., 2003b). The virus was positive in liver, spleen and lung on Day 4 and also appeared to have lower affinity for kidney, adrenal, lung, testis, lymph node, and pancreas (Baskerville et al., 1978, 1985; Bowen et al., 1978; Geisbert et al., 2003b). Mean virus titers in these organs increased progressively and reached the highest level of 10^5.5−8.6^ pfu/g on Day 6 (Geisbert et al., 2003b).

Total blood cell counts revealed marked neutrophilia and lymphopenia in the monkeys. Neutrophils and immature neutrophils increased remarkably by Day 4 (Fisher-Hoch et al., 1985; Geisbert et al., 2003b; Ebihara et al., 2011). Coincident with this process, severe lymphopenia due to lymphocyte apoptosis developed by Day 3 (Fisher-Hoch et al., 1985; Geisbert et al., 2003b). Extensive lymphocyte apoptosis, both in the vasculature and in lymphoid tissue, appears to be critical to the pathogenesis of EHF. Especially within the CD8^+^ subset, the NK cell population dropped dramatically in the early stage of infection (Geisbert et al., 2003b). Lymphocytes were not productively infected and the apoptosis was not associated with direct viral infection (Geisbert et al., 2000). However, the mechanism underlying such apoptosis is unclear. Another characteristic feature was the abnormality of platelet function preceding thrombocytopenia (Fisher-Hoch et al., 1985; Geisbert et al., 2003b). Thrombocytopenia developed between 3 and 4 days and abnormalities in coagulation parameters, including prolonged PT, aPTT, and TT appeared (Geisbert et al., 2003b; Ebihara et al., 2011). Examination of coagulation parameters revealed that decreased protein C coagulation inhibitor activity due to excessive consumption triggered severe coagulopathy as indicated by prolonged coagulation times and decreased fibrinogen levels (Ebihara et al., 2011).

The NHP model has been proven to be valuable in providing new information regarding filoviral pathogenesis. EBOV spreads from the initial infection site via monocytes/macrophages and DCs to regional lymph nodes, likely via lymphatics, and to liver and spleen through the blood stream. Tissue macrophages, including Kupffer cells, DCs, and fibroblastic reticular cells become infected with EBOV at this stage. EBOV activates DCs by upregulating expression of tumor necrosis factor-related apoptosis-inducing ligand (TRAIL), which is expressed on DCs and mediates their cytotoxic activity (Geisbert et al., 2003b). Such overexpression of TRAIL is enhanced by overexpression of IFN-α in NHPs infected with EBOV and triggers lymphocyte apoptosis. Monocytes/macrophages infected with EBOV release various soluble factors including proinflammatory cytokines to recruit additional target cells to areas of infection. As disease progresses, increased levels of oxygen free radicals (e.g., nitric oxide) released from virus-infecting macrophages at the inflammatory sites trigger apoptosis of NK cells. The lymphocyte apoptosis caused by TRAIL and nitric oxide interferes with the innate immune response, resulting in escape of EBOV infection from mounting an adaptive response. Coagulation abnormalities are not the direct result of EBOV replication-induced cytolysis of endothelial cells, but are likely triggered by immune-mediated mechanisms (Geisbert et al., 2003d,e). Extensive viral replication leads to increased levels of additional proinflammatory cytokines, notably IL-6, which triggers the coagulation irregularities. This is probably through upregulation of tissue factor expression/release from virus-infected monocytes/macrophages. Tissue factor works as the primary cellular inhibitor of coagulation protease cascades. Activation of the coagulation cascade induces the fibrinogenic and fibrinolytic pathways and finally leads to DIC, hemorrhagic shock, thrombosis-related organ failure and death (Arai et al., 2000).

Monkey species-specific disease features of the pathogenesis of EBOV infection were observed, not only in the development of cutaneous rash but also in the impairment of the clotting systems of African green monkeys and baboons infected with EBOV. In African green monkeys, fibrin thrombosis was generalized in all visceral organs, while in baboons hemorrhages were prominent in visceral organs, most notably in the liver and spleen (Ryabchikova et al., 1999; Ignatiev et al., 2000). Genetic differences, even among the same animal species, and the origin of a species may influence disease presentation and progression.

The dose and species of challenge virus affects the progression of disease. Intramuscular inoculation of cynomolgus macaques with 10^3^ pfu of EBOV produced a 100% lethal infection, with deaths occurring 6–7 days post-infection (Geisbert et al., 2002). When the challenge dose was lowered to 10 pfu, uniform lethality was still achieved, but deaths occurred 9–12 days post-infection (Geisbert et al., 2003c). Viremia was demonstrated as early as 24 h after subcutaneous infection of rhesus macaques with a high infectious dose (10^5^ pfu) of EBOV. In rhesus and cynomolgus macaques infected with 10^3^ pfu of EBOV, viremia is first detected by Day 3 after infection. SUDV causes slower disease progression than EBOV and it has been reported that some monkeys infected with SUDV did not die and recovered from the illness (Ellis et al., 1978; Bowen et al., 1980; Fisher-Hoch et al., 1992). One of four rhesus macaques infected with SUDV died on Day 12 but the other macaques survived and remained normal thereafter (Ellis et al., 1978). The dead macaque had small numbers of virus particles in the liver but no virus particles were found in the kidney, spleen, heart, lung, and brain. The liver, lung, and spleen from EBOV infected macaques, which were moribund and killed on Day 6 contained large numbers of virions. The apparent limitation of viral replication in the liver of SUDV-infected host and the contrasting widespread involvement of liver and other organs such as the spleen and lung of EBOV-infected host are similar in patients and macaques. RESTV, which is considered not to be virulent to humans, is clearly less pathogenic than EBOV and SUDV in African green monkeys and cynomolgus macaques (Fisher-Hoch et al., 1992; Jahrling et al., 1996). Only 5 of 16 monkeys infected with EBOV or SUDV survived, whereas 11 of 15 monkeys infected with RESTV survived (Fisher-Hoch et al., 1992). Viremia, clinical signs (temperature rise, anorexia, depression, or evidence of disturbed hemostasis), serum chemistry changes (elevated aspartate aminotransferase and lactate dehydrogenase activities) and pathological changes (necrosis of hepatocytes, adrenalcytes, and lymphoid elements of the spleen and prominent fibrin thrombi and fibrin precipitation) in RESTV infected monkeys developed slower and/or milder than those observed in monkeys infected with EBOV and/or SUDV.

Most human cases are thought to occur by direct contact with blood and/or body secretions from patients or animal cadavers. Aerosol transmission among humans has not been reported. However, evidence of intercage transmission of RESTV was observed in the 1989–1990 epizootic cases of RESTV in the Hazleton facility in Reston, Virginia, and demonstration of high concentrations of ebolavirus in nasal secretions and alveoli in experimental infection implicated the potency of aerosol transmission of ebolavirus (Baskerville et al., 1978, 1985; Bowen et al., 1978; Jahrling et al., 1990, 1996; Dalgard et al., 1992; Jaax et al., 1995; Miranda et al., 1999, 2002). Furthermore, the rhesus macaques experimentally challenged with aerosolized EBOV developed the same disease as macaques infected parenterally (Johnson et al., 1995). Regardless of the route of infection (intramuscular, subcutaneous, conjunctival, and aerosol injections), NHPs are highly susceptible to EBOV infection (Baskerville et al., 1978; Bowen et al., 1978, 1980; Ellis et al., 1978; Baskerville et al., 1985; Fisher-Hoch et al., 1985, 1992; Johnson et al., 1995; Jaax et al., 1996; Jahrling et al., 1996; Davis et al., 1997; Ryabchikova et al., 1999; Ignatiev et al., 2000; Geisbert et al., 2003b,e).

## Vaccines

### Inactivated whole virion

The development of filovirus vaccines has been performed based on inactivated whole virion preparations. About half of the rhesus macaques or African green monkeys treated were protected against homologous MARV challenge, when formalin- or gamma-inactivated whole MARV virions were used as vaccine candidates (Ignat'ev et al., 1991; Ignatyev et al., 1996). Vaccination with formalin-inactivated EBOV virions protected 4 of 5 hamadryas baboons (Mikhailov et al., 1994), while other studies suggested that inactivated virus did not induce sufficient immunity to protect baboons against a lethal challenge (Chupurnov et al., 1995). Furthermore, vaccination with gamma-irradiated EBOV virions alone or in a form of liposomes containing lipid A failed to protect cynomolgus macaques against lethal infection (Geisbert et al., 2002). Overall, these vaccine candidates based on inactivated virions did not confer sufficient protection in NHP models. Furthermore, these vaccines are unlikely to be used in humans due to safety risk of incomplete inactivation. However, these results promoted the development of an alternative vaccine platform, such as DNA-based vaccines, recombinant viral vector, or virus-like particles as described below and Table 2.

**Table 2:** **Efficacy of vaccines in animal models of filovirus infection**.

**Vaccine**	**Viral protein including vaccines**	**Species tested**	**Strategy**			**Challenge virus**	**Survival rate (%)**	**References**
			**Dose/Schedule**		**Route**			
VEEV	MARV Musoke GP or NP	Guinea pig	10^6^ FFU, 2 or 3 doses		sc	GP-adapted MARV Musoke	100	Hevey et al., 1998
	MARV Musoke GP or GP + NP	NHP	10^7^ FFU, 3 doses			MARV Musoke	100	
	MARV Musoke NP						67	
	EBOV NP or GP + NP	Mouse	2 × 10^6^ IU, 2 doses			mouse-adapted EBOV	100	Pushko et al., 2001
	EBOV GP						90	
	EBOV GP or GP + NP	Guinea pig		GP-adapted EBOV	100	
	EBOV NP						20	
	EBOV GP, NP or GP + NP	NHP	10^7^ FFU, 3 doses			EBOV	0	Geisbert et al., 2002
AdV	GPs of MARV (Musoke and Ci67) and RAVV	Guinea pig	5 × 10^7−8^ PFU, 2 doses		sc	MARV (Musoke or Ci67) or RAVV	100	Wang et al., 2006a
	MARV Angola GP	NHP	10^11^ PU, 1dose		im	MARV Angola	100	Geisbert et al., 2010a
	EBOV GP	Mouse	10^8^ PFU, 2 doses		sc	mouse-adapted EBOV	100	Wang et al., 2006b
	EBOV GP + NP	NHP	2 × 10^12^ particles, 1 or 2 doses		im	EBOV	100	Sullivan et al., 2003
	GPs of EBOV and SUDV, MARV (Musoke and Ci67) and RAVV + NP of EBOV and MARV Musoke		4 × 10^10^ PFU, 2 doses			EBOV, SUDV or MARV (Musoke or Ci67)	100	Swenson et al., 2008a
DNA	MARV Musoke or RAVV GP	Guinea pig	10 μg, 3 or 4 doses with RIBI aadjuvand		sc	GP-adapted MARV Musoke	100	Riemenschneider et al., 2003
	MARV Musoke GP	NHP	20 μg, 3 doses			MARV Musoke	67	
	MARV Angola GP		4 mg, 4 doses		im	MARV Angola	100	Geisbert et al., 2010a
	EBOV GP	Mouse	0.5 μg, 4 doses			mouse-adapted EBOV	78	Vanderzanden et al., 1998
			0.5 μg, 1 dose and 1.5 μg, 3 or 4 doses				100	
	EBOV GP or NP	Guinea pig	500 μg, 4 doses			GP-adapted EBOV	100	Xu et al., 1998
DNA + AdV	DNA: GPs of EBOV, SUDV and TAFV + EBOV NP AdV: EBOV GP	NHP	4 mg of DNA, 3 doses and boosted with 10^10^ PFU of AdV		im	EBOV	100	Sullivan et al., 2000
	DNA: MARV Angola GP AdV: MARV Angola GP		4 mg of DNA, 3 doses and boosted with 10^11^ PU of AdV			MARV Angola	100	Geisbert et al., 2010a
HPIV3	EBOV GP or GP + NP	Guinea pig	10^5.3^ PFU		in	GP-adapted EBOV	100	Bukreyev et al., 2006
	EBOV GP, GP + NP, or GP + GM-CSF	NHP	4 × 10^6^ TCID50, 1 dose		in and intracheally	EBOV	83	Bukreyev et al., 2007
	EBOV GP		2 × 10^7^ TCID50, 2 doses				100	
HPIV3/ΔHN-F	EBOV GP	Guinea pig	4 × 10^5^ PFU, 1 dose		in	GP-adapted EBOV	100	Bukreyev et al., 2009
VSV	MARV Musoke GP	NHP	2 × 10^7^ PFU, 1 dose	28 day before infection	im	MARV (Musoke or Angola) or RAVV	100	Daddario-Dicaprio et al., 2006a
			10^7^ PFU, 1 dose	28 or 141 d before infection^a^		MARV Musoke and Popp	100	Jones et al., 2005
	EBOV GP	Mouse	2 × 10^5^ PFU, 1 dose	24 h before infection	ip	mouse-adapted EBOV	100	Feldmann et al., 2007
				30 mpi			100	
				24 hpi			100	
		Guinea pig		24 h before infection		GP-adapted EBOV	67	
				1 hpi			83	
				24 hpi			50	
		NHP	10^7^ PFU, 1 dose	28 day before infection	im	EBOV	100	Jones et al., 2005
			10^7^ PFU, 1 dose	262 day before infection^b^		SUDV	25	
	EBOV GP + SUDV GP + Musoke GP		3 × 10^7^ PFU, 1 dose	28 day before infection		EBOV, SUDV, TAFV or MARV Musoke	100	Geisbert et al., 2009
	MARV Musoke GP		2 × 10^7^ PFU, 1 dose	24 hpi		MARV Musoke	83	Geisbert et al., 2010c
				48 hpi			33	
			1 × 10^7^ PFU, 1 dose	20–30 mpi			100	Daddario-Dicaprio et al., 2006b
	EBOV GP		2 × 10^7^ PFU, 1 dose, 20–30 mpi			EBOV	50	Feldmann et al., 2007
	SUDV GP					SUDV	100	Geisbert et al., 2008
VLP	MARV Musoke GP + VP40 produced in 293T^c^	Guinea pig	50 μg, 3 doses with RIBI adjuvant		im	GP-adapted MARV (Musoke or Ci67) or RAVV	100	Swenson et al., 2008b
		NHP	1 mg, 3 doses with QS-21 adjuvant			MARV (Musoke or Ci67) or RAVV	100	
	EBOV GP + VP40 + NP produced in 293T^c^	Mouse	50 μg, 2 doses, with QS-21 adjuvant			Mouse-adapted-EBOV	100	Warfield et al., 2007a
	EBOV GP + VP40 produced in 293T^c^		10 μg, 3 doses		im or ip		100	Warfield et al., 2003
	EBOV GP + NP + VP40 produced in 293T^c^	NHP	250 μg, 3 doses, with RIBI adjuvant		im	EBOV	100	Warfield et al., 2007b
	EBOV GP + VP40 produced in insect cells^d^	mouse	50 μg, 2 doses			Mouse-adapted-EBOV	100	Sun et al., 2009
			10 μg, 3 doses				83	
	EBOV GP + VP40 + NP produced in insect cells^d^		10-50 μg, 2 doses, with QS-21 adjuvant				100	Warfield et al., 2007a

a Cynomolgus macaques were immunized by intramuscular injection with a single dose of VSVΔG expressing MARV Musoke GP and subsequently challenged on Day 28 after immunization by intramuscular injection with MARV Musoke strain. The immunized macaques, which were protected from the lethal MARV challenge, were rechallenged with MARV Popp strain 113 days after initial challenge (141 days after immunization).

b Cynomolgus macaques were immunized by intramuscular injection with a single dose of VSVΔG expressing EBOV GP and subsequently challenged on Day 28 after immunization by intramuscular injection with EBOV. The macaques protected from the lethal EBOV challenge were rechallenged with SUDV 234 days after initial challenge (262 days after immunization).

c 293T cells were cotransfected with plasmid vectors encoding GP and VP40 (and NP) of EBOV or MARV. The VLPs were collected and purified from the cell supernatants.

d The VLPs were produced by use of recombinant baculovirus constructs expressing GP and VP (and NP) of EBOV or MARV from coinfected insect cells.

Abbreviations *FFU* *focus-forming unit* *GP-adapted* *guinea pig-adapted* *IU* *infectious unit* *PFU* *plaque-forming units* *PU* *particle units* *sc* *subcutaneously* *im* *intramuscularly* *in* *intranasally* *ip* *intrapenitoneally* *mpi* *minutes post-infection* *hpi* *hours post-infection* *dpi* *days post-infection.*

### Venezuelan equine encephalitis virus replicon

Venezuelan equine encephalitis virus (VEEV) replicons that express either GP or NP of MARV Musoke protected guinea pigs from viremia and death caused by GP-adapted MARV challenge (Hevey et al., 1998). Cynomolgus macaques administrated with MARV Musoke GP-expressing VEEV replicons alone or in combination with NP were also protected from lethal infection with the homologous Musoke strain, but not from heterologous RAVV (Hevey et al., 1998; Falzarano et al., 2011). additionally, vaccination with NP alone prevented death but not disease onset in two of three monkeys and allowed all animals to become viremic. For EBOV, EBOV GP-expressing VEEV replicons, alone or in combination with EBOV NP-expressing VEEV replicons, protected mice, and guinea pigs from lethal infection, whereas immunization with EBOV NP-expressing VEEV replicons alone protected mice but not guinea pigs (Pushko et al., 2001). Furthermore, vaccination with recombinant VEEV, expressing EBOV GP, NP, or both GP and NP, failed to protect cynomolgus macaques from a lethal EBOV infection (Geisbert et al., 2002). One recent study produced different results, whereby a VEEV-based vaccine was fully protective in cynomolgus macaques against EBOV, SUDV, and MARV (Friedrich et al., 2012). The results obtained from these studies are inconsistent, suggesting that VEEV-based vaccine may be promising although further research is needed.

### Adenovirus-based vaccines

Adenovirus (AdV) vectors commonly used are based on serotype 5 (AdV5). A single infection of the recombinant MARV Angola GP-expressing AdV5 resulted in complete protection of cynomolgus macaques from illness and death by challenge with homologous virus (Geisbert et al., 2010a). Vaccination with a mixture of EBOV GP—expressing AdV5 and EBOV NP—expressing AdV5 have demonstrated 100% protection in cynomolgus macaques against homologous virus challenge (Sullivan et al., 2003). However, the genome insert size in this first generation AdV vector was restricted to as little as a single filovirus GP gene. The second generation AdV vector has the advantage of being able to express multiple antigens in a single construct over the first generation vector. The second generation bivalent AdV vector expressing GPs of EBOV and SUDV led to efficient induction of antibodies specific to EBOV and SUDV (Wang et al., 2006b). A trivalent AdV vector expressing MARV GPs of Ci67, Musoke and RAVV efficiently led to MARV-specific antibodies in mice and guinea pigs and showed complete protection of guinea pigs against MARV and RAVV infections (Wang et al., 2006a). Additionally, vaccination of cynomolgus macaques with second generation AdV vectors, which expressed multiple filovirus GPs of EBOV, SUDV, Ci67, Musoke, and RAVV, induced 100% protection against challenge with EBOV and SUDV and two different strains of MARV (Ci67 and Musoke; Swenson et al., 2008a). Although the AdV-based vaccines showed efficacy, the vaccines have a major obstacle: the prevalence of pre-existing immunity to AdV that may substantially limit their immunogenicity and clinical utility. It is estimated that the prevalence of antibody to AdV5 is up to 60% in the general human population and up to 85% in Africa (Schulick et al., 1997; Piedra et al., 1998). Indeed, macaques pre-immunized against AdV5 and vaccinated with EBOV GP-expressing AdV5 were not protected from lethal challenge with EBOV infection (Geisbert et al., 2010b). AdV serotype 26 and 35 segregated genetically from AdV5 exhibit lower seroprevalence in humans (Vogels et al., 2003; Abbink et al., 2007; Mast et al., 2010). Therefore, AdV serotype 26 and 35 vectors with expression of EBOV or SUDV GPs have been generated and the protective efficacy examined by using the NHP model, but these vectors failed to protect cynomolgus macaques against lethal EBOV challenge (Geisbert et al., 2011).

### DNA

The plasmid coding the DNA of GP from MARV Musoke or RAVV demonstrated efficacy in protection of guinea pigs and cynomolgus macaques against lethal infection of each homologous strain (Riemenschneider et al., 2003). All of the guinea pigs vaccinated three or four times with DNA vaccines were aviremic and appeared healthy. In cynomolgus macaques, four of six monkeys immunized with 3 doses of DNA vaccine encoding Musoke GP were protected from homologous challenge with MARV Musoke (Riemenschneider et al., 2003). In a report of DNA vaccines encoding GP of MARV Angola strain, the 4 vaccination doses resulted in protection of all four vaccinated monkeys, but three of the four monkeys showed symptoms and/or lymphopenia (Geisbert et al., 2010a). A combination vaccine regimen (3 times injection with DNA and boost with recombinant Angola GP-expressing AdV vector) protected the monkeys from lethal infections but two of the four monkeys showed rash or lymphopenia. A single inoculation with AdV vaccine induced optimal immune responses to eliminate symptoms and death by itself. These data suggest that DNA vaccines do not optimally control MARV infection (Geisbert et al., 2010a). However, three-plasmid DNA vaccines encoding EBOV GP, SUDV GP, and EBOV NP were evaluated in a phase I trial as safe and immunogenic in humans (Martin et al., 2006). The EBOV DNA vaccine also protected mice and guinea pigs against a lethal challenge (Vanderzanden et al., 1998; Xu et al., 1998; Martin et al., 2006). In one study, cynomolgus macaques, which received 3 injections of DNA vectors encoding EBOV GP, SUDV GP, TAFV GP, and EBOV NP, were boosted with recombinant EBOV GP-expressing AdV (Sullivan et al., 2000). All four monkeys vaccinated survived and showed no symptoms of EBOV infection. This prime-boost strategy provided a sufficient immune response to clear the virus efficiently.

### Human parainfluenza virus

In an outbreak of RESTV in the Hazleton facility in Reston, Virginia, aerosol transmission between NHPs may have occurred (Jahrling et al., 1990; Dalgard et al., 1992; Miranda et al., 1999, 2002). To address the assumed aerosol transmission of filovirus, a vaccine that induces a strong immune response in the respiratory tract was developed. Human parainfluenza virus type 3 (HPIV3), a common respiratory virus, was modified as a form of vaccine vector and used for development of a vaccine against EBOV. The HPIV3 vectors, which express EBOV GP or EBOV GP together with NP, protected guinea pigs and rhesus macaques against EBOV challenge (Bukreyev et al., 2006, 2007). In guinea pigs, a single intranasal inoculation with HPIV3 expressing EBOV GP or both GP and NP showed complete protection against signs of illness and death (Bukreyev et al., 2006). The rhesus macaques were immunized with a single dose of EBOV GP-expressing HPIV3, or EBOV GP and NP-expressing HPIV3, through a combined intranasal and intratracheal inoculation. Five of six monkeys immunized with the HPIV3 based vaccine survived and four of six monkeys did not show any clinical illness (Bukreyev et al., 2007). Two doses of intranasal immunizations showed greater efficacy, including complete protection of all three rhesus macaques against clinical illness and death. However, HPIV3 may not be effective as a vaccine vectors in humans, since HPIV3 is a common childhood pathogen and the majority of the population have pre-existing immunity to HPIV3. To overcome the problem of pre-existing immunity, a chimeric HPIV3, where both HPIV3 surface proteins, HN and F, were deleted and replaced with EBOV GP was developed (Bukreyev et al., 2009). A single immunization with the vaccine completely protected guinea pigs against a lethal infection. It was shown that the HPIV3 based vaccine, which expressed EBOV GP, was immunogenic equally among HPIV3-naïve and HPIV3 antibody-positive subjects and effective when vaccinated twice. However, pre-existing HPIV3-specific immunity in rhesus macaques reduced the replicative capacity of the HPIV3-based vaccine in the respiratory tract (Bukreyev et al., 2010). Nevertheless, this study indicated that the vaccination induced an appropriate antibody response.

### Vesicular stomatitis virus

A vaccine to resolve the problem of pre-existing immunity utilized the recombinant vesicular stomatitis virus (VSV) vector, which expresses filovirus GP. VSV is mainly a veterinary pathogen and human infection with VSV is rare and not associated with disease in humans. A single intramuscular vaccination of cynomolgus macaques with recombinant VSV with expression of MARV Musoke GP elicited complete protection against a high dose (10^3^ pfu) intramuscular challenge with both homologous Musoke strain and heterologous Popp strain, Angola strain, and RAVV (Jones et al., 2005; Daddario-Dicaprio et al., 2006a). For EBOV, a single immunization of cynomolgus macaques with recombinant VSV vector, which expresses EBOV GP, also elicited complete protection against EBOV challenge (Jones et al., 2005). The surviving macaques from lethal EBOV infection were re-challenged with heterologous SUDV, but the cross-protection was not observed (Jones et al., 2005). Administration of the EBOV GP-expressing VSV vaccine through the oral or intranasal route completely protected cynomolgus macaques from EBOV challenge (Qiu et al., 2009). A blended vaccine consisting of equal amounts of 3 different VSV vectors, which expresses GP of each of EBOV, SUDV, and MARV, generated complete protection of cynomolgus macaques against challenges with EBOV, TAFV, and MARV (Geisbert et al., 2009). Macaques vaccinated with the blended vaccine followed by challenge with SUDV showed mild clinical sign of illness including fever, lymphopenia, and mild anorexia, and the macaques recovered from illness. Importantly, none of the macaques vaccinated with the blended vaccine succumbed to a filovirus challenge. The efficacy of the recombinant VSV vaccine has been evaluated as a post-exposure prophylaxis for filovirus infections. Administration of recombinant VSV with MARV Musoke GP expression to rhesus macaques shortly after a homologous high-dose MARV challenge resulted in complete protection of all subjects from clinical illness and death (Daddario-Dicaprio et al., 2006b). Furthermore, administration of recombinant MARV Musoke GP-expressing VSV at 24 and 48 h following infection resulted in protection of 83 and 33% of rhesus macaques, respectively (Geisbert et al., 2010c). When recombinant EBOV GP-expressing VSV were administrated to mice 24 h prior to challenge, and 1 and 24 h post-challenge, all treated mice survived (Feldmann et al., 2007). In guinea pigs treated with EBOV GP-expressing VSV at 24 h prior to challenge, and 1 or 24 h post-challenge, the survival rates were 67, 83, and 50%, respectively. It was also demonstrated that post-exposure vaccination with the recombinant VSV GP vectors for EBOV and SUDV in rhesus macaques was effective against challenge with homologous viruses, although the protection rate was dependent on the species of ebolavirus. The survival rates of the EBOV- or SUDV-infected monkeys were 50 and 100%, respectively (Feldmann et al., 2007; Geisbert et al., 2008).

### Virus-like particle

Virus-like particle (VLPs), which mimic authentic virions structurally but do not contain infectious genetic material, are non-infectious and safer than replicating vaccines. The efficiency of a MARV vaccine consisting of VLPs with MARV Musoke GP and VP40 was assessed in guinea pig and cynomolgus macaque models (Swenson et al., 2008b). The guinea pigs and monkeys immunized three times with MARV-Musoke VLPs with RIBI or QS-21 adjuvant were challenged with Musoke strain, Ci67 strain, or RAVV. All guinea pigs and eight monkeys were protected from death and clinical illness following the lethal challenge, except for a single monkey. The monkey challenged with RAVV, which is the most genetically distinct strain of marburgvirus, developed minor signs of disease without detectable viremia. For ebolavirus, mice vaccinated with EBOV VLP in the presence or absence of adjuvant were protected from lethal EBOV infection in a dose-dependent manner (Warfield et al., 2003, 2007a; Sun et al., 2009). Furthermore, the efficacy of the EBOV VLP, which consists of EBOV GP, NP, and VP40 was evaluated in cynomolgus macaques (Warfield et al., 2007b). All five monkeys that received three injections of the EBOV VLPs with RIBI adjuvant were completely protected against EBOV challenge.

There are some other vaccine candidates, including an EBOV lacking VP30 (which encodes the essential transcription factor), an Fc portion of a human IgG fused to EBOV-GP, a bean yellow dwarf virus-derived replicon system, and a cytomegalovirus-based vaccine encoding an EBOV NP CTL epitope (Halfmann et al., 2009; Konduru et al., 2011; Phoolcharoen et al., 2011; Tsuda et al., 2011a). However, the immunogenic efficacy of these vaccines has only been confirmed in the rodent models and further studies are needed to evaluate the protective efficacy and safety in NHPs.

## Treatments

### Recombinant nematode anticoagulant protein C2

Coagulation abnormalities are one of the most prominent hallmarks of filovirus infection. It has been suggested that tissue factor plays an important role in triggering the hemorrhagic complications in NHPs infected with filoviruses (Geisbert et al., 2003d). Overexpression of tissue factor that performs as the primary cellular inhibitor of the coagulation protease cascades is one of the causes of DIC and thrombosis-related organ failure. The effect of blocking the pathway leading from the complex of activated factor VII and tissue factor to thrombin was examined in filovirus infection. Recombinant nematode anticoagulant protein c2 (rNAPc2), which directly inhibits factor VII and tissue factor, provided partial post-exposure protection to rhesus macaques infected with filovirus (Geisbert et al., 2003a, 2009). In rNAPc2-treated rhesus macaques, the mean survival time (11.7 days) was longer than that in untreated control monkeys (8.3 days) and 33% of EBOV-infected macaques survived. In MARV Angola-infected rhesus macaques treated with rNAPc2, 1 of 6 (17%) monkeys survived and the mean survival time for the five dead monkeys was significantly prolonged compared with that of the untreated control monkeys. rNAPc2 demonstrated a clear improvement in terms of survival rate and an increase in mean survival time in a normally 100% lethal model of filovirus infection.

### Recombinant human activated protein C

Activated protein C (APC) is generated from the protein C, which is a vitamin K-dependent plasma protein and inactivates factors V and VIII to down-regulate thrombin generation. It has been reported that circulating levels of protein C were rapidly and significantly reduced in cynomolgus macaques and rhesus macaques during EBOV infections, because the protein C might be produced in the liver, which is a main target of filovirus infection (Geisbert et al., 2003a). In rhesus macaque models, administration of recombinant human APC (rhAPC) at 30–60 min after challenge and continuing for 7 days, protected 2 of 11 (18%) monkeys against lethal EBOV infection (Hensley et al., 2007). The mean survival time in the rhAPC-treated monkeys was prolonged compared with the untreated monkeys (Hensley et al., 2007).

### Phosphorodiamidate morpholino oligomer

Phosphorodiamidate morpholino oligomers (PMOs) inhibit targeted gene translation by steric blockage of ribosomal assembly. A combination of EBOV-specific PMOs targeting sequences of viral mRNAs for the VP24, VP35, and RNA polymerase L protected rodents in both pre- and post-exposure therapeutic regimens (Warfield et al., 2006). In rhesus macaque models, treatment with a combination of the PMOs of VP24, VP35, and L from 2 days prior to EBOV challenge through Day 9 of the infection protected 3 of 4 (75%) rhesus macaques against lethal infection (Warfield et al., 2006). Furthermore, it was demonstrated that the antiviral potency of PMOs could be enhanced by chemical modification, either by conjugating PMOs with peptides or by introducing positively charge to the PMOs (PMO*plus*™, Avi BioPharma, Inc.; Swenson et al., 2009). Subsequently, PMO*plus* targeting EBOV VP24 and VP35 or MARV Musoke VP24 and NP showed significant protection of mice and guinea pigs against lethal challenge with EBOV and MARV Musoke, respectively (Warren et al., 2010). AVI-6002 PMO*plus* against both EBOV VP24 and VP35, and AVI-6003 PMO*plus* against MARV VP24 and NP, were developed and tested for treatment efficacy using NHP models. These PMOs, delivered 30–60 min post-exposure, protected 62.5% of rhesus macaques against lethal EBOV infection and 100% of cynomolgus macaques against MARV Musoke infection (Warren et al., 2010). AVI-6002 and AVI-6003 are currently in phase I clinical trials.

### RNA interference

RNA interference (RNAi) inhibits gene expression to the extent that their function is abrogated through a highly regulated enzyme-mediated process. It was demonstrated that small-interfering RNA (siRNA) down-regulated various MARV mRNA transcripts, resulting in a significant decrease in viral protein production and subsequent viral release *in vitro* (Fowler et al., 2005). Furthermore, siRNA targeting the EBOV RNA polymerase L protein formulated in stable nucleic acid-lipid particles (SNALPs) completely protected guinea pigs when administered shortly after a lethal EBOV infection (Geisbert et al., 2006). In rhesus macaques, a combination of siRNA targeting the EBOV L, VP24, and VP35 were formulated in SNALPs and administrated to the monkeys. Two of three monkeys, which were treated four times with siRNA at 30 min, 1, 3, and 5 days after challenge, survived lethal infection. Furthermore, all four monkeys treated seven times at 30 min, 1–6 days after challenge survived (Geisbert et al., 2010d).

### Therapeutic efficacy in the mouse model and *in vitro*

In the mouse model, administration of recombinant mannose-binding lectin and hexaamminecobalt (III) chloride showed efficacy in protecting against EBOV infections (Michelow et al., 2011). Mannose-binding lectin targets diverse microorganisms for phagocytosis and complement-mediated lysis by binding specific surface glycans. Hexaamminecobalt (III) chloride is a complex of a cobalt (III) ion surrounded by six ammonia ligands in a full octahedral coordination. Furthermore, by high-throughput screening, some compounds such as FGI-103, FGI-106, and NSC 62914 (a reactive oxygen species scavenger), were identified to have high antiviral activity against filoviruses (Aman et al., 2009; Warren et al., 2010). Some other substances, for example inhibitors of heat-shock protein 90 and Niemann-Pick C1, showed antiviral activity *in vitro* (Smith et al., 2010; Cote et al., 2011). As mentioned above (Table 3), several candidates are discussed as therapeutic agents for Ebola and Marburg HFs, but no licensed therapeutics are yet available (Friedrich et al., 2012).

**Table 3:** **Efficacy of post-exposure treatment in animal models of filovirus infection**.

**Treatment**	**Mechanism/target viral protein**		**Species tested**	**Strategy**			**Challenge virus**	**Survival rate (%)**	**References**
				**Dose**	**Route**	**Dose schedule**			
rNAPc2	Blocks TF: FVIIa mediated activation of factor X		NHP	30 μg/kg bw	sc	10 mpi and administraion daily for 14 days	EBOV	33	Geisbert et al., 2003a
						24 hpi and administraion daily for 8 days		33	
						10 mpi and administraion daily for 14 days	MARV Angola	17	Geisbert et al., 2007
APC	Anti-thrombotic: cleaves and inhibits coagulation cofactors FVIIIa and Fva		NHP	2 mg/m^2^/h	iv	30–60 mpi and administration for 7 days	EBOV	18	Hensley et al., 2007
PMO	Targets viral mRNA to block transcription	EBOV VP24,VP35 and L	Mouse	500 μg	ip	twice at 24 h and 4 h before infection	mouse-adapted EBOV	100	Warfield et al., 2006
						single dose at 24 hpi		100	
			Guinea pig			single dose 24 h before infection	GP-adapted EBOV	>25	
						single dose 24 hpi		25–50	
						single dose 96 hpi		50–75	
			NHP	12.5–200 mg	im	2 day before challenge and administration for 9 days	EBOV	75	
PMO plus		EBOV VP24 and VP35 (AVI-6002)	NHP	40 mg/kg bw	sc and ip	30–60 mpi and administration daily for 10 or 14 days	EBOV	63	Warren et al., 2010
				28 or 40 mg/kg bw	iv	30-60 mpi and administration daily for 14 dpi		60	
				4 mg/kg bw				0	
				16 mg/kg bw				20	
		MARV Musoke VP24 and NP (AVI-6003)	NHP	30 or 40 mg/kg bw	sc and ip		MARV Musoke	100	
				40 mg/kg	sc or iv			100	
				30 mg/kg	iv			100	
				7.5 or 15 mg/kg				60	
siRNA	Targets viral mRNA to block transcription	EBOV L	guinea pig	PEI-mixed, 8 mg/kg	ip	3 h before infection and 24, 48 and 96 hpi	GP-adapted EBOV	25	Geisbert et al., 2006
				SNALP-formulated, 1 mg/kg		1, 24, 48, 72, 96, 120 and 144 hpi		60^a^	
				SNALP-formulated, 0.75 mg/kg				100	
		EBOV L, VP24 and VP35	NHP	SNALP-formulated, 2 mg/kg	iv	30 mpi, 1, 3 and 5 dpi	EBOV	66	Geisbert et al., 2010d
		EBOV L, VP24 and VP35				30 mpi, 1, 2, 3, 4, 5 and 6 dpi		100	

a Two of five guinea pigs received the siRNAs using the SNALP delivery systems died but the death could not be attributed to viral replication.

Abbreviations *bw* *bodyweight* *sc* *subcutaneously* *iv* *intravenously* *ip* *intrapenitoneally* *im* *intramuscularly* *mpi* *minutes post-infection* *hpi* *hours post-infection* *dpi* *days post-infection.*

## Conclusions

Significant progress has been made in developing animal models, including mice, guinea pigs, hamsters and NHPs, for EHF and MHF. The NHPs are the most feasible model, because they are the only animals that are lethally infected with non-adapted virus isolates and the pathophysiology is close to that demonstrated in patients. The rodent models need serial passages of original filoviruses in rodents for acquiring lethal infection capacity and they have limited value, because the disease course in rodents differs from that demonstrated in humans and NHPs. However, the rodent models are the first choice for preliminary studies to explore vaccines and therapeutic agents, because of their ease to handling. The newly developed Golden hamster model will also be used for studies on pathogenesis and evaluation of efficacy of candidate vaccines and therapeutics because they show manifestations similar to those of patients and NHPs, including severity of coagulopathy that is lacking in mouse and guinea pig models. Among the candidate vaccines so far developed, recombinant VSV-based vaccines against EHF and MHF are confirmed to be effective in mouse, guinea pig, Golden hamster, and NHP models, and are the only platform with the potential to prevent lethal infection, especially via both vaccine and post-exposure treatment (Jones et al., 2005; Daddario-Dicaprio et al., 2006b; Feldmann et al., 2007; Geisbert et al., 2008, 2009, 2010c; Qiu et al., 2009; Tsuda et al., 2011b). Furthermore, the VSV have been used as a treatment following a recent laboratory exposure (Tuffs, 2009). Further research is needed to develop vaccines with sufficient long-term efficacy by single-dose vaccination, because expensive and time-consuming vaccinations may pose difficulties due to logistical and financial problems in developing countries, where EHF and MHF are endemic. Neither licensed vaccines nor therapeutic agents are available so far. The development of vaccines and therapeutic testing using the animal models has only recently begun to progress. We hope that further research facilitates progress toward elucidating the disease pathophysiology and developing prophylactic and therapeutic measures against EHF and MHF.

### Conflict of interest statement

The authors declare that the research was conducted in the absence of any commercial or financial relationships that could be construed as a potential conflict of interest.
